# *Deqi* Is Double-Faced: The Acupuncture Practitioner's and the Subject's Perspective

**DOI:** 10.1155/2015/635089

**Published:** 2015-11-22

**Authors:** Chang Shik Yin, Younbyeong Chae, O-Seok Kang, Seung-Tae Kim, Dae-Hyun Hahm, Ji-Yeun Park, Hyejung Lee, Hi-Joon Park

**Affiliations:** ^1^Acupuncture and Meridian Science Research Center, Kyung Hee University, Seoul 130-701, Republic of Korea; ^2^Department of Acupuncture and Meridian, College of Korean Medicine, Kyung Hee University, Seoul 130-701, Republic of Korea; ^3^Division of Meridian and Structural Medicine, School of Korean Medicine, Pusan National University, Pusan 626-813, Republic of Korea; ^4^College of Korean Medicine, Daejeon University, Daejeon 34520, Republic of Korea

## Abstract

*Background*. While therapeutic acupuncture perception (*deqi*) has recently been investigated only for the subject's perception, classical acupuncture discussed acupuncture perception for both the practitioner and the subject. The aim of this study was to explore the practitioner's and the subject's acupuncture perception during acupuncture. *Methods*. Explorative crossover study to quantitatively document acupuncture perception of both the practitioner and the subject. Eighty-one participants acted as a practitioner or a subject. The practitioner's and the subject's acupuncture perceptions were collected using self-report type checklists. Acupuncture needles were inserted to LI4 or ST36, adopting a four-phase method: insertion into shallow, middle, and deep depths, followed by twirling manipulation. Pain, transmission, dullness, and soreness feelings of the subject and thick, tangled, solid, and empty feelings of the practitioner were analyzed for their correlation. *Results*. The practitioner's and the subject's perception showed a significant correlation. Acupuncture perception varied over four phases of needling, with a tendency to be rated higher when inserted deep. Perception for LI4 was generally higher than those for ST36. *Conclusion*. The practitioner's acupuncture perception was successfully documented and analyzed in relation to the subject's acupuncture perception and different needling conditions.

## 1. Introduction

Acupuncture belongs to a stimulation-based therapy. Acupuncture has been suggested to act as neuromodulating inputs to the nervous system [1], and the effects of it may safely be referred to be sensory-driven effects. Acupuncture modulates specific brain structures [2] and electrical activities [3]. Sensory input to the human body is usually generated by a filiform needle. Considering that a needle is manually operated, the sensory input may vary according to the practitioner's manual art.

Acupuncture practitioners try to generate an optimal input for the improvement of the patient's health by developing and performing the sophisticated art of it, which includes palpation, insertion, manipulation, and withdrawal of the needle. The question remains whether the administration of a predetermined set of procedures to a patient is appropriate or not. In other words, predetermined protocols may not represent real acupuncture because real acupuncture necessitates incessant feedback from patients and adjustment of needling. Furthermore, it is debatable whether there are any reliable markers for the practitioner to get consistent information about the progression and reaction of the patient following the applied procedures. Diverse sensations are induced by acupuncture, and differential brain areas are activated accordingly [2, 4]. A practitioner needs instant and continuous feedback to monitor his or her own interventional procedures and adjust them to an optimal one. Constant monitoring of the subject gives feedback to the practitioner, and in that way the practitioner modulates the needling procedure. Ways of subject monitoring include asking, observing, and palpating.

*Deqi*, a therapeutic acupuncture perception, has been asserted to be a useful surrogate of the activity of acupuncture treatment [5–7] and a criterion to determine the appropriateness of acupuncture stimulation [4, 8]. However, it appears not clear whether it is the patient, practitioner, or both who experience* deqi* [9]. Traditional definition of* deqi*, or obtaining qi (得氣), is two-way one: (1) the subject's perceptions reflecting proper physiological changes [10–12] including heaviness, numbness, soreness, distension, or even sensation like refreshing, relieving, or warm (2) together with the practitioner's feeling of tenseness around the needle [13]. The subject's perception may be documented by asking the subject. The practitioner's perception/feeling is instantly obtained from the tissues by the use of the practitioner's fingers and the needle. Palpable changes in the tissues around the acupoint may be transmitted to the practitioner through the needle he or she holds and inserts. This art could be developed through clinical experience and, when sophisticated to a degree, provides the practitioner with additional information distinguishable from possibly ambiguous verbal answer of the subject. However, previous researches on* deqi* have only dealt with the subjective feeling of the subject.

We report for the first time on the* deqi* phenomenon perceived from the practitioner's perspective and show a correlation between practitioner's and the subject's perceptions.* Deqi* data were obtained from both the acupuncture practitioner and the subject and analyzed in relation to the insertion depths, needle manipulation, and stimulated acupoints.

## 2. Materials and Methods

### 2.1. Study Design

This study implemented a crossover design trying to observe (1) changes in acupuncture perception in relation to differential acupoints, needling depths, and manipulations and (2) correlations between the practitioner's and the subject's perception.

All the study procedures were performed at the Kyung Hee University. The study protocol was reviewed and approved by a local ethics committee. Participants were college students who attended an acupuncture practicum class. All the participants were genetically homogenous Koreans and had previously experienced acupuncture both as a practitioner and as a receiver. Only the data obtained from those who provided an informed consent were collected and analyzed. Data obtained from 81 out of 112 participants were included in the analysis (Table 1).

### 2.2. Main Outcome Measure

Subject's acupuncture perception was documented using a modified Korean version of the acupuncture perception scale [6, 7] which was of self-report type and comprised 20 items. Each item was rated on a 7-point Likert scale, where “0” represented no such feeling and “6” represented an unbearably intense feeling. Average ratings of (1) total 20 items (electric, hot, and the following 18 items), (2) 9 pain domain items (ache, sharp, stinging, hurting, pricking, intense, penetrating, shocking, and burning), (3) 3 transmission domain items (radiating, pulsing, and spreading), (4) 4 dullness domain items (dull, heavy, numb, and pulling), and (5) 2 soreness domain items (tingling and throbbing) [14] were calculated and stored for later analysis.

The practitioner's acupuncture perception was documented using a Korean version of the acupuncture practitioner's perception scale which was of self-report type and comprised four items: thick, tangled, solid, and empty feeling (Table 2). These items were chosen based on a traditional clinical description [14]. Acupuncture needle practitioners rated their presumable feeling related with the tissues adjacent to the tip of the needle they have just inserted using a 7-point Likert scale (range 0–6), where “0” represented no such feeling and “6” represented an unbearably intense feeling. In addition to the four ratings, average ratings of the total four items were calculated and stored for later analysis.

### 2.3. Acupuncture Needling and Experimental Procedures

Every two participants were instructed to pair up as an acupuncture practitioner and an acupuncture subject. Disposable needles used were 0.25 mm (diameter) × 40 mm (length) and made of stainless steel (Dongbang Acupuncture, Boryeong, Republic of Korea). Acupoints LI4 and ST36 were chosen as they are frequently used in acupuncture manipulation training considering safety, accessibility, and feasibility to induce or experience acupuncture perception [15]. Acupuncture needling was performed through four phases. A practitioner penetrated the skin of the acupoint LI4 on the nondominant side with a needle using a guide tube and then slowly (about 1 cm per 6 seconds) inserted the needle vertically into a shallow depth of about 3 mm (Phase 1). A practitioner inserted the needle further 3 mm into a middle depth (Phase 2) and then further 3 mm into a deep depth (Phase 3), followed by twirling the needle 180 degrees in alternate directions once a second for 10 seconds (Phase 4). Each participant practiced this type of insertion and manipulation method beforehand. Insertion depth was verified using a guide tube or ruler. This kind of slow advancement or retraction of the needle tip below the skin is a frequent way of training in Korean acupuncture manipulation. It is usual that this slow and soft manipulation is not painful, because most part of the pain in acupuncture needling involves penetration of the skin and strenuous manipulation. The practitioner and subject repeatedly filled in the form of relevant acupuncture perception scale during 30 seconds of interval which was placed after each phase of acupuncture needling without knowing the others' rating. The same procedure was repeated for the acupoint ST36 on the nondominant side. Then, the practitioner and the subject changed their roles and the whole procedure was repeated.

### 2.4. Statistical Analysis

Acupuncture perception ratings over four phases of stimulation for each acupoint were compared using one way analysis of variance followed by Duncan* post hoc* multiple comparisons. Paired sample *t*-test was performed to compare ratings for LI4 and ST36. Correlation between practitioner's rating and subject's rating was explored by Pearson correlation coefficients. SPSS statistical software (version 17 for Windows) was used. Significance level was set at 0.05 by two-tailed test.

## 3. Results

### 3.1. Acupuncture Perception over Three Needling Depths and after Twirling Manipulation at Acupoint LI4

**Figure 1:**
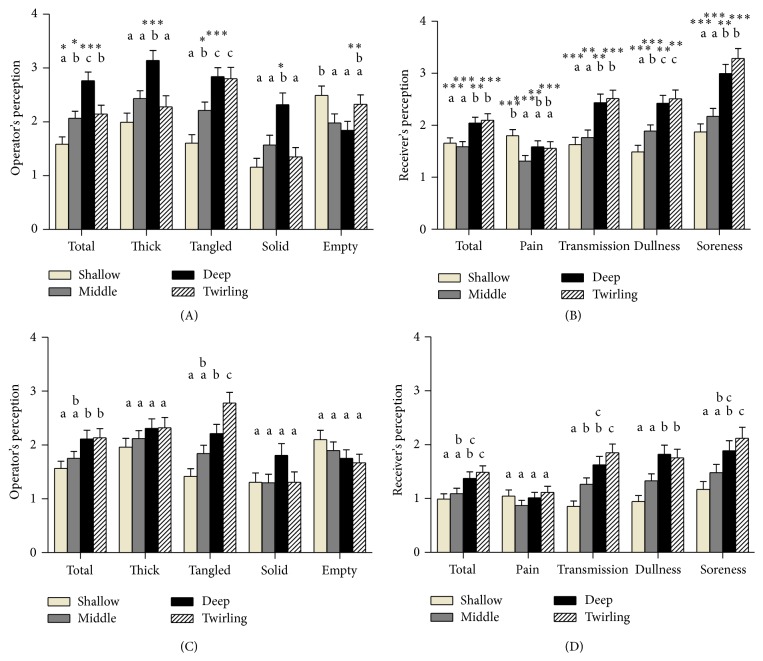
Intensity of acupuncture perception. Practitioners (*n* = 81) of an acupuncture needle (A, C) or subjects (*n* = 81) of an acupuncture stimulation (B, D) subjectively rated the intensity of feeling using a 7-point Likert scale (range 0–6) for the acupoint LI4 (A, B) or ST36 (C, D). Acupuncture stimulation and rating were performed through four sequential phases: (1) insertion into shallow depth (approximately 0.3 cm), (2) advancement into moderate depth (approximately 0.6 cm), (3) advancement into deep depth (approximately 0.9 cm), and (4) twirling once a second for 10 seconds. Values are represented as mean ± standard error. Superscript letters (a, b, and c) represent homogenous subset groups by Duncan* post hoc* multiple comparisons following one way analysis of variance of ratings over four phases. ^*∗*^ *p* < 0.05, ^*∗∗*^ *p* < 0.01, and ^*∗∗∗*^ *p* < 0.001 by paired sample *t* test between ratings for LI4 and those for ST36.

Practitioner's ratings over four phases of needle insertion were significantly different for total, thick, tangled, solid (*p* < 0.001), and empty feeling (*p* < 0.05). Subject's ratings over four phases of needle insertion were significantly different for transmission, dullness, soreness (*p* < 0.001), total (*p* < 0.01), and pain feeling (*p* < 0.05).* Post hoc* comparisons revealed homogenous subsets. As the needle insertion depth changed from upper to middle and to lower position, practitioners rated the total, thick, tangled, and solid feeling as higher and rated the empty feeling as lower (Figure 1(A)). As the needle insertion depth changed from upper to middle and to lower position, subjects rated the total, transmission, dullness, and soreness feeling as higher and the pain feeling as lower (Figure 1(B)).

### 3.2. Acupuncture Perception over Three Needling Depths and after Twirling Manipulation at Acupoint ST36

Practitioner's ratings over four phases of needle insertion were significantly different for tangled (*p* < 0.001) and total feeling (*p* < 0.05). Subject's ratings over four phases of needle insertion were significantly different for transmission, dullness (*p* < 0.001), total, and soreness feeling (*p* < 0.01).* Post hoc* comparisons revealed homogenous subsets. As the needle insertion depth changed from upper to middle and to lower position, practitioners rated the total and tangled feeling as higher (Figure 1(C)). As the needle insertion depth changed from upper to middle and to lower position, subjects rated the total, transmission, dullness, and soreness feeling higher (Figure 1(D)).

### 3.3. Comparison of Acupuncture Perception between LI4 and ST36

The ratings of both the practitioners and the subjects were higher for LI4 compared to S36 for all feelings. The thick and solid feeling was significantly different only at deep depth, with the empty feeling significantly different only after the twirling manipulation. Subject's feelings were significantly different in all four phases of acupuncture stimulation (Figure 1).

### 3.4. Correlation between Practitioner's and Subject's Ratings for LI4

Practitioner's rating and subject's rating for all four phases of acupuncture stimulation (*n* = 384) showed a significant correlation. When the ratings of each phase were analyzed separately (*n* = 81), subject's rating of pain and dullness showed significant correlation with practitioner's rating in phase 3 (deep position) and phase 4 (twirling), respectively (Table 3).

### 3.5. Correlation between Practitioner's and Subject's Ratings for ST36

Practitioner's rating and subject's rating for all four phases of acupuncture stimulation (*n* = 384) showed a significant correlation. When the ratings of each phase were analyzed separately (*n* = 81), subject's rating of pain showed significant correlation with practitioner's rating in phase four (twirling) (Table 4).

## 4. Discussion

Main finding of this study includes the correlation between the acupuncture practitioner's and the subject's perception. The practitioner's facet of acupuncture needle perception is first reported here. Variations in acupuncture perception were also observed in relation to the insertion depths and twirling manipulation, which were reflected in both the practitioner's and the subject's perceptions.

Traditional training of classical style of acupuncture involves the development of clinical art of palpation, even in locating an acupoint [16]. In chapter 78 of an acupuncture book* Classic of Difficult Issues* (難經), it is mentioned that “those with an expertise in acupuncture rely on the left hand and those without rely on the right hand.” Acupuncture needling needs the constant help of the left or the nondominant side. Palpation has been for long at the heart of clinical art [17], and manual or hands-on healing arts such as chiropractic, applied kinesiology [18], intraoral balancing appliance therapy [19], and acupuncture have always put emphasis on this skill which requires both science and art. Palpation is much more than just pressure (algometry), and it involves proprioception, motion, and tension [20].

Unlike the verbal response of the subjects, palpation provides instant and rather objective additional information about the subjects' state and response of the tissues palpated. While inserting and manipulating the needle, tissue state and response around the inserted acupoint can be felt through the needle. This may belong to a type of palpation in classical acupuncture practice. This palpation is performed through the needle being inserted into the tissue of acupoint and the fingers grasping the needle. This feeling is another source of constant feedback information other than verbal response of the subjects and should be developed through clinical experience and training. The practitioner's objective feeling is the other side in the definition of “*deqi*” as the subject's subjective feeling. Qi arrival (氣至), a feeling sensed by practitioners during needling [21], is another example describing palpable tissue changes related with presumable “qi movement” or other therapeutic responses within the subject's body.

Until now,* deqi* has been investigated only on the acupuncture subject's side. It is also commonplace in modern acupuncture clinics that the physician asks the patients about their own subjective perception. In other words, acupuncture physicians came to rely more on the patients' verbal saying and less on their own art of palpation.

This study dealt with the acupuncture perception of the practitioner and found that the practitioner's perception showed a correlation with the normal subject's perception, meaning that the practitioner's acupuncture perception felt through the needle may well be utilized as a way of monitoring the tissue state or the response of the subjects. This monitoring skill of the practitioner through the needle may be practiced and further developed to get more information on the subject's current status. The practitioner's acupuncture perception could be considered as a kind of palpation where, in a sense, the acupuncture needle is an extension of palpating fingers. It possibly delivers clinically useful information, although the reliability or validity of palpation is still controversial [22, 23]. It correlated mainly with pain and dullness and did not seem to have constant correlation with a specific kind of subject's acupuncture perception (Tables 3 and 4).

Consistent with previous reports [24], the subject's acupuncture perceptions for LI4 were generally rated higher than those for ST36. This was also the case for the practitioner's acupuncture perception. As the needle is inserted deeper, the acupuncture perception of both the subject and the practitioner tended to be rated higher.

The results and discussions of this study may inevitably accompany limitations; (1) the study population may not be large enough to represent the whole population including both the healthy ranging and the unhealthy ranging from the younger to the elder, (2) the correlation coefficients is not fairly high, (3) the needling depths and manipulation were not rigidly controlled, and (4) the observation in this study was limited to the possible correlation between acupuncture practitioner's and subject's perceptions without considering the correctness of the precise acupoint location and the possible clinical effect of acupuncture needle manipulation. These issues may have to be further reviewed and addressed in later studies.

The findings of this study may present clinically useful subjects of discussion. Is it worth to further develop the clinical art of palpation in acupuncture training courses and clinical practice? Considering that subjective and objective acupuncture perception may vary according to the insertion depths and needle manipulation, optimal stimulation intensity and type may have to be differentially found even when acupuncture treatment is administered to the same acupoint. Given that acupuncture perception intensity and type varied in relation to the stimulated acupoint and the stimulation method, one rigid criterion of acupuncture perception may not well fit into clinical reality. More flexible criteria or flexibly applied variations of one preset criterion might be necessary.

In this study, there may have been random variations in the insertion depth and twirling of the needle over different practitioners and insertion phases, partly because of the practitioner's skill and partly because of the innate nature of manual manipulation. Possible variations in the insertion depth and twirling of the needle may be within reasonable limit and may not be a problem interpreting the result of this study, considering that the aim of this study was not to contrast acupuncture perception with needling parameters but to contrast a practitioner's perception with a subject's perception. In this study, a practitioner's perception and a subject's perception under the same parameters of needling were recorded and were compared. However, further researches with a more robust design considering a crossover or parallel design, diverse needling parameters, needling skills, and participants such as an experienced clinical acupuncturists group and an acupuncture naive age- and health-matched control group may show more definite data with minimized possibilities of random variations.

In conclusion, the practitioner's acupuncture perception was successfully documented and analyzed in relation to the subject's acupuncture perception and different needling conditions.

## Figures and Tables

**Table 1:** Demographic data of the participants.

Gender (*n*), female/male	20/61
Age (years)	23.6 ± 3.3
Handedness (*n*), right/left	81/0

Data are presented as number (*n*) or mean ± standard deviation.

**Table 2:** Acupuncture practitioner's perception scale.

Instruction: check the intensity of the feeling of the tissue felt through the needle and needling fingers (0: none, 6: strongest imaginable).							
Thick feeling	0	1	2	3	4	5	6
Tangled feeling	0	1	2	3	4	5	6
Solid feeling	0	1	2	3	4	5	6
Empty feeling	0	1	2	3	4	5	6

**Table 3:** Correlation coefficients between the practitioner's and the subject's perceptions over four needling phases for the acupoint LI4.

	Practitioner's perception				
	Total	Thick	Tangled	Solid	Empty
Subject's perception					
Total	0.215^*∗∗∗*^ (P4: 0.233^*∗*^)	0.173^*∗∗∗*^ (P1: 0.260^*∗*^)	0.184^*∗∗*^	0.182^*∗∗*^ (P4: 0.242^*∗*^)	−0.039
Pain	0.194^*∗∗∗*^ (P3: 0.269^*∗*^)	0.183^*∗∗*^	0.134^*∗*^ (P3: 0.247^*∗*^)	0.170^*∗∗*^ (P3: 0.227^*∗*^)	−0.011
Transmission	0.119^*∗*^	0.084	0.122^*∗*^	0.091	0.041
Dullness	0.191^*∗∗*^ (P4: 0.286^*∗*^)	0.128^*∗*^ (P4: 0.274^*∗*^)	0.172^*∗∗*^	0.179^*∗∗*^ (P4: 0.309^*∗∗*^)	−0.117^*∗*^
Soreness	0.180^*∗∗*^	0.142^*∗*^	0.200^*∗∗∗*^	0.112^*∗*^	−0.053

^*∗*^ *p* < 0.05, ^*∗∗*^ *p* < 0.01, and ^*∗∗∗*^ *p* < 0.001 by Pearson correlation analysis over four phases (*n* = 384) or each one of four needling phases (*n* = 81, value in parentheses). P represents a needling phase number: (1) insertion into shallow depth (approximately 0.3 cm), (2) advancement into moderate depth (approximately 0.6 cm), (3) advancement into deep depth (approximately 0.9 cm), and (4) twirling once a second for 10 seconds.

Four-phase method: insertion into shallow (P1), middle (P2), and deep depths (P3), followed by twirling manipulation (P4).

**Table 4:** Correlation coefficients between the practitioner's and the subject's perceptions over four needling phases for the acupoint ST36.

	Practitioner's perception				
	Total	Thick	Tangled	Solid	Empty
Subject's perception					
Total	0.197^*∗∗*^ (P4: 0.228^*∗*^)	−0.006	0.174^*∗∗*^	0.182^*∗∗*^	0.150^*∗∗*^
Pain	0.239^*∗∗*^ (P4: 0.333^*∗∗*^)	0.225^*∗∗*^ (P1: 0.268^*∗*^) (P4: 0.281^*∗*^)	0.185^*∗∗*^ (P4: 0.276^*∗*^)	0.204^*∗∗*^ (P4: 0.319^*∗*^)	0.000
Transmission	0.118^*∗*^	0.091	0.143^*∗∗*^	0.072	0.029 (P2: 0.264^*∗*^)
Dullness	0.129^*∗*^	0.111^*∗*^	0.150^*∗∗*^	0.072	−0.043
Soreness	0.171^*∗∗*^	0.138^*∗*^	0.181^*∗∗*^	0.122^*∗*^	−0.013

^*∗*^ *p* < 0.05, ^*∗∗*^ *p* < 0.01, and ^*∗∗∗*^ *p* < 0.001 by Pearson correlation analysis over four phases (*n* = 384) or each one of four needling phases (*n* = 81, value in parentheses). P represents a needling phase number: (1) insertion into shallow depth (approximately 0.3 cm), (2) advancement into moderate depth (approximately 0.6 cm), (3) advancement into deep depth (approximately 0.9 cm), and (4) twirling once a second for 10 seconds.

Four-phase method: insertion into shallow (P1), middle (P2), and deep depths (P3), followed by twirling manipulation (P4).
